# Prevention of lymphedema after extended pelvic lymphadenectomy using diosmin, bromelain, melilotus, and hesperidin: results from a prospective randomized study (PRELYNE trial)

**DOI:** 10.1007/s11701-026-03816-5

**Published:** 2026-08-19

**Authors:** Francesca Montanaro, Greta Pettenuzzo, Sarah Malandra, Alessandro Veccia, Antonio Benito Porcaro, Alessandra Gozzo, Alberto Bianchi, Riccardo Rizzetto, Francesco Artoni, Sonia Costantino, Maria Angela Cerruto, Riccardo Bertolo, Alessandro Antonelli, Collaborators, Elisabetta Muscolino, Gioacchino Giambalvo, Alessio Valota, Martina Dolci, Paolo Casa, Filippo Caudana, Dario Brunello

**Affiliations:** 1 University of Verona, Verona, Italy; 2 Department of Urology, Azienda Ospedaliera Universitaria Integrata Verona, Verona, Italy

## Abstract

Extended pelvic lymph node dissection (ePLND) during robot-assisted radical prostatectomy (RARP) may impair lymphatic drainage, causing lower-limb and genital lymphedema with a potential negative impact on postoperative recovery and quality of life. This prospective randomized study evaluated whether an oral formulation containing diosmin, bromelain, melilotus, and hesperidin (Meliven Top^®^) could reduce postoperative lymphatic morbidity after RARP with ePLND. One hundred patients undergoing RARP with ePLND for prostate cancer were randomized 1:1 to standard postoperative care alone or standard care plus oral Meliven Top^®^ for one month. Lower-limb volumes were assessed preoperatively and at 1 and 3 months postoperatively using circumferential measurements and truncated-cone volume estimation. Secondary endpoints included genital swelling, genital pain, and lymphocele formation. Multivariable ANCOVA models adjusted for baseline limb volume, body mass index, and number of lymph nodes removed were applied.Ninety-eight patients completed follow-up. No significant differences in lower-limb volumetric changes were observed between groups at 1 or 3 months. However, genital pain at 1 month was significantly lower in the treatment group (2% vs. 18%; *p* = 0.008), and persistent genital swelling at 3 months was absent in treated patients compared with controls (0% vs. 8%; *p* = 0.041). Lymphocele rates were similar between groups. In conclusion, Meliven Top^®^ did not significantly reduce postoperative lower-limb volume changes after RARP with ePLND, but it was associated with improved genital symptom recovery. Larger studies with longer follow-up are warranted.

**Clinical trial registration** This study was not registered in a public clinical trial registry.

**Supplementary Information:**

The online version contains supplementary material available at 10.1007/s11701-026-03816-5.

## Introduction

Extended pelvic lymphnode dissection (ePLND) during robot-assisted radical prostatectomy (RARP) is routinely considered in patients with intermediate- and high-risk prostate cancer, according to an estimation of nodal involvement [1], mainly to achieve a correct staging of disease. The staging benefit of ePLND, however, should be weighed against procedure-related morbidity, particularly concerning complications depending on the impairment of lymphatic system function [2].

Among lymphatic events symptomatic lymphocele is the most frequently reported [3], but lower-limb and genital edema, resulting from the disruption of pelvic lymphatic drainage and the accumulation of protein-rich interstitial fluid, is underreported and under-investigated although representing, at various degrees, the common sequela of ePLND. Lymphedema can potentially severely impact patient’s quality of life as genital or lower-limb swelling may affect hygiene, mobility, urinary recovery, sexual rehabilitation, and patient perception of recovery, in addition to a frank deterioration of self-image perception.

A few studies have been focused on postoperative lymphedema after RARP with ePLND, also because to be captured requires a limb-volume and genital swelling prospective and objective, pre- and post-operative assessment [4]. This gap in the scientific literature appears particularly relevant when considering that, conversely, the same event involving the upper limbs has been extensively investigated in patients undergoing lymphadenectomy for breast cancer [5].

Oral agents targeting edema, microcirculation, and inflammatory pathways has an attractive rationale and can be easily administered during routine postoperative care, given the lack of interaction with other medications and regular healing. Meliven Top^®^ is an oral formulation containing diosmin, bromelain, melilotus, and hesperidin. The present randomized trial evaluated its role after RARP with ePLND. The primary endpoint was postoperative lower-limb volume change; secondary endpoints were genital swelling, genital pain, and lymphocele formation.

## Materials and methods

### Study design and population

After Institutional Review Board Approval (4080CESC), patients scheduled to undergo RARP with ePLND for histologically confirmed prostate cancer at the Urology Unit of AOUI Verona were screened for eligibility. Eligibility criteria, perioperative management, intervention details, and follow-up assessments were defined before enrollment.

This was an exploratory, hypothesis-generating randomized study. No formal sample size calculation or power analysis was performed before study initiation. The sample size was pragmatically determined by the availability of the study supplement acquired exclusively for research purposes, allowing the enrollment of 100 consecutive eligible patients.

The patients were enrolled consecutively, with equal allocation to the intervention and control arms. All patients underwent surgery and postoperative care according to the same institutional pathway, except for the assigned oral treatment. Baseline variables included age, body mass index (BMI), cardiovascular disease, diabetes mellitus, hypertension, baseline prostate-specific antigen (PSA), MRI PI-RADS score, pathological Gleason score, pathological tumor stage, pathological nodal status, and number of lymph nodes removed. Self-reported adverse events were collected.

### Eligibility criteria

Patients were eligible if they were aged 18 year or older, of either sex, scheduled to undergo pelvic lymphadenectomy, and able to provide written informed consent before enrollment.

Exclusion criteria were age < 18 year, severe obesity, previous dependent edema documented by clinical history or physical examination, systemic or local diseases known to be associated with dependent edema, use of medications known to be associated with dependent edema, previous preoperative radiotherapy, and refusal to provide written informed consent.

Patients could be withdrawn during the study in case of consent withdrawal, onset of a systemic or local condition associated with dependent edema, or initiation of medications associated with dependent edema.

### Randomization and intervention

Patients were randomized in a 1:1 ratio to standard postoperative care alone or standard care plus oral Meliven Top^®^. The allocation sequence was generated before study initiation using Research Randomizer, which provided a numerical randomization list for 100 participants. Patients were assigned sequentially according to this list, with planned allocation of 50 patients per group.

Oral Meliven Top^®^ was administered at a dose of one tablet daily, starting within the first postoperative day and continued for one month. Treatment adherence was not systematically monitored using pill counts, patient diaries, or other objective measures. Apart from administration of the study product, perioperative care- including the ePLND template, postoperative drain management, thromboprophylaxis, rehabilitation, clinical surveillance, and follow-up schedule- followed the same standardized institutional protocol in both study arms. No additional preventive interventions specifically targeting lymphedema, including manual lymphatic drainage, compression garments, physiotherapy, structured exercise programs, or other decongestive treatments, were routinely prescribed during the study period. Because the control group did not receive a placebo, participants and treating physicians were aware of treatment allocation; however, physicians performing lower-limb volumetric assessments were blinded to the assigned study arm.

### Lymphedema assessment

Lower-limb lymphedema was evaluated through standardized anthropometric measurements based on circumferential limb assessment and truncated-cone volume estimation. Total lower-limb length was measured from the greater trochanter to the malleolus. Five circumferential measurements were obtained for each limb at predefined anatomical intervals. Consecutive limb portions were modeled as truncated cones, and segmental volumes were combined to obtain thigh, shank, and total lower-limb volumes for each side [6, 7]. Because standardized assessment protocols for lower-limb lymphedema after pelvic lymph node dissection are lacking, circumference-based limb volumetry using the truncated-cone method was selected as an objective and reproducible approach. Similar volumetric methods have been widely adopted in prospective studies of breast cancer-related lymphedema and provide a practical framework for serial postoperative assessment. The 1- and 3-month evaluations were chosen to characterize early postoperative lymphatic morbidity and its initial evolution.

No predefined volumetric threshold was used to categorize patients as having established lower-limb lymphedema.

Measurements were performed at baseline, 1 month, and 3 months after surgery, by physicians blinded to treatment allocation. Genital swelling and genital pain were assessed clinically at the same time points. Genital pain and genital swelling were recorded as binary clinical variables (present/absent) during each postoperative visit.

Lymphocele occurrence was recorded by clinical and radiological (ultrasound) assessment during postoperative evaluations.

### Statistical analysis

Descriptive statistics were used to summarize baseline and follow-up variables. Continuous variables were reported as mean (standard deviation) or median [interquartile range], according to distribution. Categorical variables were expressed as absolute numbers and percentages. Baseline comparisons were performed to evaluate the balance achieved by randomization using appropriate tests for continuous and categorical variables (Wilcoxon rank-sum test, chi-square test). In addition to absolute postoperative volumes, percentage changes in thigh and shank volumes between 1 and 3 months were calculated and compared between treatment arms.

To estimate treatment effects on postoperative limb volumes, an analysis of covariance (ANCOVA) using a multivariable linear regression model was performed, separately for each segmental and whole-limb outcome at 1 and 3 months. The model included treatment group as the main factor, along with baseline limb volume, BMI, and the number of lymph nodes removed as covariates. Robust standard errors were used to improve model reliability, and Holm correction was applied for multiple comparisons [8]. Treatment effects were expressed as adjusted differences relative to control, with corresponding 95% confidence intervals. A two-sided p-value < 0.05 was considered statistically significant. All analyses were performed using Stata version 19.0 (StataCorp LLC, College Station, TX, USA).

## Results

Between February 2023 and September 2025, 100 patients submitted to RARP and ePLND satisfying inclusion criteria were randomized. During follow-up, one patient in the intervention arm discontinued treatment before the first postoperative month because of severe urinary incontinence and a medical recommendation to interrupt the draining regimen while continuing rehabilitative exercises. One patient in the control arm was lost at the first follow-up. The final analysis included 98 patients: 49 receiving standard postoperative care and 49 receiving standard care plus oral Meliven Top^®^.

No statistically significant differences were observed for age, BMI, cardiovascular disease, diabetes, hypertension, baseline PSA, MRI PI-RADS distribution, pathological Gleason score, tumor stage, nodal status, or number of lymph nodes removed. Baseline lower-limb lengths and segmental volumes were also comparable, supporting the validity of adjusted postoperative comparisons [Tables 1 and 2].

**Table 1:** Baseline features

	Total *N* = 98	Control group *N* = 49	Treatment group *N* = 49	*p*
**Age**				
Median [IQR]	65.5 [61–71]	64 [60–70]	67 [62–71]	0.2
**BMI**				
Median [IQR]	26 [23–28.5]	27 [24–29]	25 [23–28]	0.18
**CAD**				
N (%)	10 (11)	3 (6)	7 (14)	0.2
**Diabetes**				
N (%)	11 (11)	5 (10)	6 (12)	0.8
**Hypertension**				
N (%)	50 (51)	26 (53)	24 (49)	0.5
**PSA baseline**				
Median [IQR]	8 [6–12]	8 [5–12]	8 [6–11.5]	0.5
**PI-RADS N°1**				
N (%) ** 3** ** 4** ** 5**		_*N*=42_ 4 (9.5) 21 (50) 17 (40.5)	_*N*=44_ 3 (7) 16 (36) 25 (57)	0.17
**Gleason surgery**				
** 6** ** 7** ** 8** ** 9**		1 (2) 31 (63) 14 (29) 3 (6)	0 30 (61) 13 (27) 6 (12)	0.53
**pT**				
** T2a** ** T2b** ** T2c** ** T3a** ** T3b**		7 (13) 1 (2) 18 (37.5) 16 (33) 7 (14.5)	4 (8) 4 (8) 23 (47) 7 (14) 11 (23)	0.7
**pN+**		5 (10)	7 (14)	0.85
**Lymph nodes removed**				
Median [IQR]		20 [16–25.5]	20 [11–26]	0.62
**Lower limb lenght**				
** rt** ** lh**	89.01 (6.9) 88.88 (7.06)	88.84 (7.72) 88.76 (7.73)	89.19 (6) 89 (6.37)	0.74 0.72
Mean (s.d.)				

**Table 2:** Baseline limbs volume

	Total *N* = 98	Control group *N* = 49	Treatment group *N* = 49	p
**Segments volume - baseline (cm** ^**3**^ **)**				
1. Thigh right 2. Thigh right A. Shank right B. Shank right 1. Thigh left 2. Thigh left A. Shank left B. Shank left	3895.8 (924.2) 3650.7 (762.5) 1918.8 (380) 1657.5 (352.9) 3794.5 (883.7) 3581.2 (729.1) 1901.1 (397.5) 1640.5 (377.3)	3904.2 (826) 3634.4 (713.7) 1926.1 (319.2) 1663.8 (274) 3781.9 (805.3) 3577.9 (683.1) 1914.7 (332.1) 1645.8 (289.2)	3887.5 (1021.7) 3667.0 (815.6) 1911.4 (435.6) 1651.2 (420.1) 3807.1 (964) 3584.4 (779.5) 1888.1 (456.8) 1635.2 (451.6)	0.93 0.83 0.85 0.86 0.89 0.96 0.75 0.89
**Thigh volume - baseline (cm** ^**3**^ **)**				
Right	7546.5 (1670.2)	7538.5 (1522.6)	7554.6 (1821.7)	0.96
Left	7375.7 (1593.6)	7359.9 (1465.8)	7391.4 (1727.2)	0.92
**Shank volume - baseline (cm** ^**3**^ **)**				
Right	5232.9 (2394.7)	5309.3 (2223.7)	5156.5 (2575.2)	0.75
Left	3541.6 (764.9)	3559.8 (609.1)	3523.3 (900.5)	0.81
Mean (s.d.)				

**Fig. 1:**
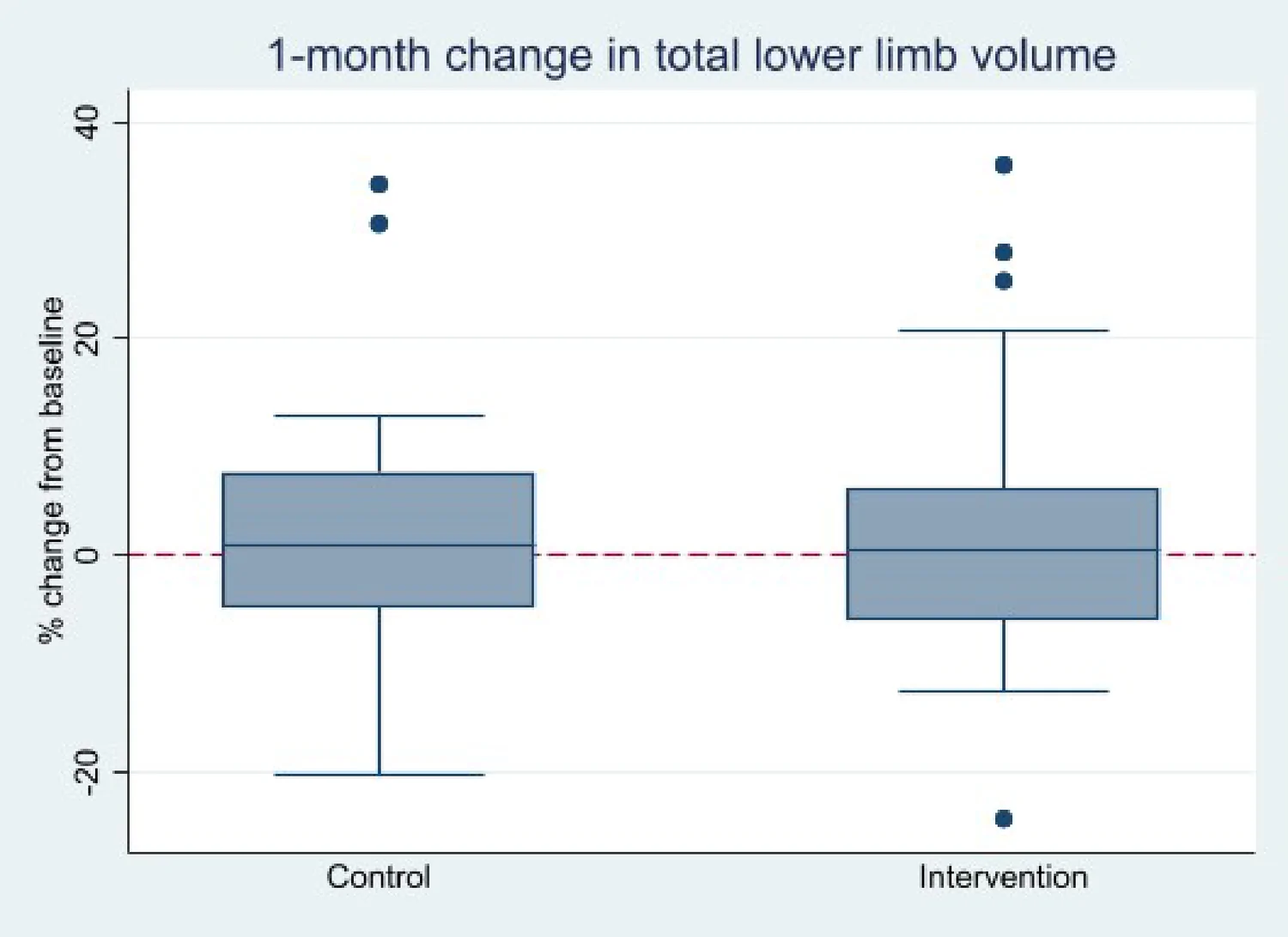
Percentage change in total lower-limb volume from baseline at 1 month after robot-assisted radical prostatectomy with extended pelvic lymph node dissection. Boxplots compare the control and Meliven Top® groups. The central line represents the median, boxes the interquartile range (IQR), whiskers extend to 1.5 × IQR, and dots indicate outliers. The dashed horizontal line represents no change from baseline (0%). No significant difference in postoperative lower-limb volume change was observed between groups

**Fig. 2:**
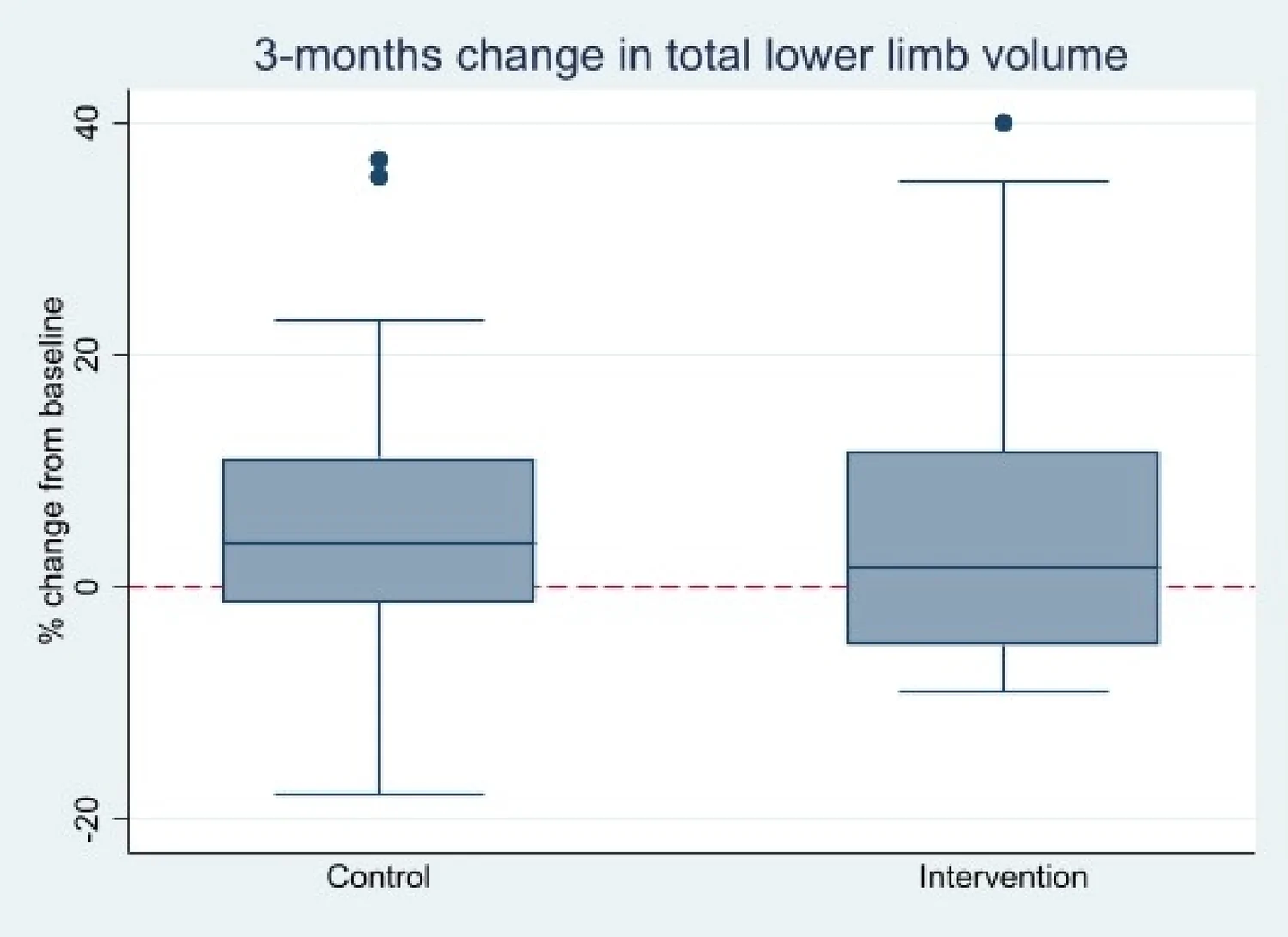
Percentage change in total lower-limb volume from baseline at 3 months after robot-assisted radical prostatectomy with extended pelvic lymph node dissection. Boxplots compare the control and Meliven Top® groups. The central line represents the median, boxes the interquartile range (IQR), whiskers extend to 1.5 × IQR, and dots indicate outliers. The dashed horizontal line represents no change from baseline (0%). No significant difference in postoperative lower-limb volume change was observed between groups

After adjustment for baseline limb volume, lower-limb volumetric outcomes were comparable between groups at both 1 and 3 months. No statistically significant adjusted differences favoring the treatment arm were observed across right or left thigh, right or left shank, or whole-limb analyses. At 3 months, the adjusted mean whole right-limb volume was 11623.79 cm3 in controls and 11673.65 cm3 in the treatment group, with an adjusted difference of 49.86 cm3 (95% CI −545.26 to 644.98; Holm-adjusted *p* = 0.87). For the whole left limb, the corresponding adjusted means were 11451.99 and 11400.38 cm3, with an adjusted difference of −51.61 cm3 (95% CI −614.22 to 510.99; Holm-adjusted *p* = 0.86). Consistently, percentage volume changes between 1 and 3 months did not differ between groups for the right thigh (7.13% vs. 3.86%; *p* = 0.92), left thigh (4.54% vs. 9.72%; *p* = 0.64), right shank (4.96% vs. 5.62%; *p* = 0.31), or left shank (4.19% vs. 5.73%; *p* = 0.66). Overall, the serial volumetric data showed similar lower-limb recovery trajectories in both arms. However, 1 and 3-month total lower limb volume variation showed a slight advantage for those in the intervention group, but this did not achieve statistical significance [Table 3-Figs. 1 and 2- Supplementary Tables 1-Supplementary Table 2].

**Table 3:** Adjusted 1–3 months limbs volume difference

Time point (month)	Group	Adjusted mean (cm^3^)	Adjusted difference vs. control (IC 95%)	Holm-adjusted *p*-value
**LOWER LIMB - RIGHT**				
**1**	**Control**	11135.68	-	0.34
**1**	**Treatment**	11694.44	558.7637 [−242.0669 1359.594]	
**3**	**Control**	11623.79	-	0.87
**3**	**Treatment**	11673.65	49.85797 [−545.2604 644.9763]	
**LOWER LIMB – LEFT**				
**1**	**Control**	10988.53	-	0.78
**1**	**Treatment**	10767.94	−220.59 [−21.9681 882.3706]	
**3**	**Control**	11451.99	-	0.86
**3**	**Treatment**	11400.38	−51.61272 [−614.2151 510.9896]	

No patient had genital swelling or genital pain at baseline. At 1-month, genital swelling was numerically less frequent in the Meliven Top^®^ arm than in the control arm (12% vs. 20%), although this difference did not reach statistical significance (*p* = 0.274). At 3 months, genital swelling persisted in 4 control patients (8%) but in none of the treated patients (*p* = 0.041). Genital pain showed an earlier difference: at 1 month, 1 treated patient (2%) and 9 control patients (18%) reported pain (*p* = 0.008). By 3 months, genital pain was uncommon and comparable between groups (2% in each arm; *p* = 1.000) [Table 4].

**Table 4:** Genital swelling and pain at 1–3 month

	Total *N* = 98	Control group *N* = 49	Treatment group *N* = 49	*p*
**Genital swelling**				
**Baseline**	0	0	0	1
**1 month**	16 (16)	10 (20)	6 (12)	0.27
**3 months**	4 (4)	4 (8)	0	0.041
*N* (%)				
**Genital pain**				
** Baseline** ** 1 month** ** 3 months**	0 10 (10) 2 (2)	0 9 (18) 1 (2)	0 1 (2) 1 (2)	1 **0.008** 1
**Lymphocele**	9 (9)	3 (6)	6 (12)	0.31

Postoperative lymphocele occurred in 9 patients overall. The incidence did not differ significantly between groups (6% in controls vs. 12% in treated patients; *p* = 0.31), suggesting no measurable effect of the oral regimen on lymphocele formation. No patient experienced any adverse event.

## Discussion

To the best of our knowledge, this prospective randomized study is among the few trials specifically addressing a nutraceutical supportive strategy for early lymphatic morbidity after RARP with ePLND. The main finding is that lower-limb volume trajectories during the first 3 postoperative months were comparable between groups. Although a statistically significant reduction in lower-limb volume was not observed, the serial design provides useful prospective information on the early course of limb-volume changes after pelvic lymphadenectomy.

This finding should be interpreted in the context of the mechanisms underlying post-lymphadenectomy edema. The increase in lower-limb volume after ePLND is likely multifactorial, involving interruption of deep pelvic lymphatic pathways, lymphatic leakage, postoperative inflammation, patient susceptibility, and possibly adjuvant treatment. An oral formulation may support anti-edematous and microcirculatory mechanisms but may not be sufficient to overcome the anatomical consequences of extensive pelvic lymphatic dissection during a short observation window. Moreover, the one-month duration of treatment may have been sufficient to influence early inflammatory or edematous changes but insufficient to modify the longer-term biological processes involved in lymphatic remodeling after ePLND.

The magnitude and direction of the volumetric results deserve specific consideration. Adjusted whole-limb differences at 3 months were minimal for both sides, and percentage changes between 1 and 3 months were not suggestive of a consistent treatment-related pattern. This supports the interpretation that early lower-limb recovery followed a substantially similar course in the two arms.

Several reasons may explain why a difference in lower-limb volume was difficult to detect. First, lower-limb lymphedema after pelvic surgery is not only a consequence of fluid accumulation, but also of lymphatic rerouting, inflammatory response, venous return, postoperative mobility, and individual tissue compliance [9]. Second, the early postoperative period may be characterized by transient volume fluctuations that are not necessarily indicative of established lymphedema. Third, patients with severe obesity, previous dependent edema, systemic or local conditions associated with edema, preoperative radiotherapy, or edema-related medications were excluded [10]. This design improves internal validity, but it may also reduce the number of high-risk patients in whom a preventive effect would be easier to demonstrate.

The second important finding is the favorable signal for genital symptoms. Genital swelling was numerically lower at 1 month and was absent in the treatment group at 3 months, while genital pain was significantly less frequent in treated patients at 1 month. These observations suggest that oral Meliven Top^®^ may be associated with a more favorable early genital symptom course, even though this effect was not mirrored by a measurable difference in lower-limb volume.

The clinical relevance of genital symptoms should not be underestimated. After prostatectomy, even limited genital swelling can have a disproportionate impact on the patient experience. It may interfere with hygiene, sitting, walking, clothing tolerance, voiding comfort, and early sexual rehabilitation. Pain in this anatomical district may also amplify the perception of delayed recovery, even when the surgical course is otherwise uncomplicated [11]. Therefore, a reduction in genital pain at 1 month and the absence of persistent genital swelling at 3 months in treated patients represent signals that may be meaningful from a patient-centered perspective, although they require confirmation.

The apparent discrepancy between lower-limb volumetric outcomes and genital symptoms is plausible. Lower-limb drainage involves a wider and deeper anatomical territory, whereas genital edema may represent a more localized and symptomatically evident form of postoperative lymphatic congestion [12]. Therefore, a systemic oral treatment could produce detectable differences in localized genital edema without causing measurable changes in total lower-limb volume over a short time frame.

This interpretation is also supported by the different nature of the endpoints. Limb-volume assessment captures quantitative changes in large anatomical compartments. By contrast, genital swelling and pain are clinical outcomes that may reflect local congestion, inflammation, tissue tension, and nerve sensitivity. A modest change in local edema may be clinically evident in the genital region, while the same degree of change would be diluted and difficult to capture when expressed as total thigh, shank, or whole-limb volume.

The formulation used in the present study includes components that have been investigated for their potential anti-edematous, microcirculatory, and anti-inflammatory properties. Diosmin and hesperidin are venoactive flavonoids that may influence capillary permeability, venous tone, and inflammatory pathways [13]. Bromelain has been evaluated for its anti-inflammatory and anti-edematous effects, while melilotus-containing preparations have been explored in lymphatic and venous disorders [14]. Although these mechanisms are biologically plausible, the present data should be interpreted clinically rather than mechanistically. The study was not designed to measure inflammatory biomarkers or lymphatic flow.

The third finding is the absence of a significant difference in lymphocele formation. This is not unexpected. Lymphocele is primarily a deep pelvic event related to lymphatic leakage, dead space, the extent and template of nodal dissection, sealing technique, anticoagulation, BMI, and postoperative drainage pathways. These factors are unlikely to be substantially modified by oral treatment alone. The numerically higher lymphocele rate in the intervention arm was not statistically significant and should not be overinterpreted, given the sample size.

From a surgical standpoint, lymphocele and peripheral edema should be regarded as related but distinct manifestations of lymphatic morbidity. Lymphocele reflects the accumulation of lymphatic fluid within the pelvis, whereas lower-limb or genital edema reflects impaired drainage in peripheral tissues. A supportive oral treatment may theoretically influence edema-related symptoms without modifying the formation of a pelvic collection. This distinction is important when interpreting negative lymphocele findings, because the absence of lymphocele reduction does not necessarily exclude a possible effect on symptomatic superficial or peripheral congestion.

From a clinical perspective, the present data should be interpreted as showing comparable lower-limb volumetric recovery between groups rather than a clear negative effect of the intervention. The absence of a statistically significant difference in the primary volumetric analyses may reflect the short observation window, the modest sample size, and the multifactorial nature of lymphatic recovery after ePLND. At the same time, the genital symptom findings support further investigation of Meliven Top^®^ as a supportive oral adjunct during the early postoperative phase.

The randomized design and the balance between groups strengthen the reliability of the comparisons. Baseline demographic, clinical, pathological, and limb-volume variables were comparable, reducing the likelihood that the observed symptom differences were driven by major imbalances. Moreover, the use of ANCOVA adjusted for baseline limb volume was appropriate because preoperative limb size may influence postoperative volume estimates. The application of robust standard errors and correction for multiple comparisons further supports a cautious statistical interpretation of the volumetric analyses.

However, several methodological issues should be considered when interpreting the results. Circumference-based volumetry is practical and reproducible in clinical settings, but it remains operator dependent. Small differences in measurement level, tape tension, limb position, or postoperative patient hydration may influence calculated segmental volumes. In addition, bilateral limb measurement can be challenging because there is no unaffected contralateral reference limb after pelvic lymphadenectomy. The present approach was suitable for a prospective study, but future work may benefit from standardized photographic documentation of measurement points, repeated measurements, or integration with bioimpedance and imaging-based assessment.

The choice of study endpoints was intentionally based on objective limb volumetry complemented by clinically relevant genital symptoms. Although similar volumetric assessment methods have been extensively applied in breast cancer-related lymphedema [5], standardized outcome measures for lower-limb and genital lymphedema after pelvic lymph node dissection remain poorly defined. Therefore, objective limb volumetry combined with clinically relevant genital outcomes was considered the most appropriate strategy for the present study.

Several limitations deserve mention. First, no formal sample size calculation or power analysis was performed before study initiation. As this was an exploratory, hypothesis-generating study, the sample size (100 patients) was pragmatically determined by the availability of the study supplement acquired exclusively for research purposes. Consequently, the study may have been underpowered to detect small but clinically meaningful differences in postoperative lower-limb lymphedema. Second, follow-up was limited to 3 months, whereas lymphatic morbidity may appear or persist beyond the early postoperative period. Third, treatment adherence was not systematically monitored using objective or structured methods, which prevented a reliable assessment of compliance. Fourth, because the study adopted an open-label design without placebo control, subjective outcomes such as genital pain and swelling may have been influenced by expectation and reporting bias. However, lower-limb volumetric assessments were based on objective measurements performed by physicians blinded to treatment allocation, thereby limiting detection bias for the primary outcome. Fifth, genital swelling and pain were clinically assessed rather than measured using validated patient-reported outcome measures (PROMs) or quality-of-life questionnaires [15]. Sixth, the study did not stratify outcomes by dissection template, BMI category, anticoagulation exposure, adjuvant treatment, or the number of nodes removed beyond baseline comparison.

Furthermore, the study did not include a predefined diagnostic threshold for established lower-limb lymphedema, as limb-volume changes were analyzed as continuous outcomes.

Another limitation is that the analysis focused on early outcomes. This time frame is clinically relevant because it reflects the period in which patients resume mobility, continence rehabilitation, and daily activities. Nevertheless, lymphedema may evolve over a longer period, and early postoperative edema may resolve or progress to persistent lymphatic dysfunction. A longer follow-up could clarify whether the genital symptom benefit is transient, whether delayed lower-limb edema emerges, and whether early symptoms identify patients at higher risk for subsequent chronic lymphatic morbidity.

Future studies should include larger cohorts, longer follow-up, adherence monitoring, patient-reported outcomes, and, when feasible, complementary lymphatic assessment. Stratified analyses may help identify patients at higher risk of lymphatic morbidity and those most likely to benefit from supportive oral interventions.

In addition, future protocols should define separate endpoints for objective limb volume, genital edema, pain, quality of life, and lymphocele. This distinction would avoid combining different biological phenomena under a single broad definition of lymphatic morbidity. Trials should also consider clinically meaningful thresholds for volume change, rather than relying only on statistical significance. Such an approach would be particularly relevant when the sample size is limited and when small numerical changes may or may not translate into patient-perceived benefit.

## Conclusions

In patients undergoing RARP with ePLND, changes in lower-limb volume during the first 3 postoperative months were comparable between standard care and standard care plus oral Meliven Top^®^. Percentage changes in thigh and shank volumes between 1 and 3 months were not significantly different between groups. Conversely, oral Meliven Top^®^ was associated with a favorable genital symptom profile, with significantly lower pain at 1 month and absence of persistent genital swelling at 3 months. Larger studies with longer follow-up and patient-centered outcomes are warranted to clarify the role of Meliven Top^®^ in postoperative lymphatic recovery.

## Supplementary Information

Below is the link to the electronic supplementary material.

Supplementary Material 1

Supplementary Material 2

## Data Availability

No datasets were generated or analysed during the current study.

## Declarations

### Permission to reproduce material from other sources

No material from previously published sources requiring permission for reproduction has been included in this manuscript.
