# Supplementary material for: Prevention of lymphedema after extended pelvic lymphadenectomy using diosmin, bromelain, melilotus, and hesperidin: results from a prospective randomized study (PRELYNE trial)
Source: J Robot Surg. 2026 Aug 19;20(1):856. doi: 10.1007/s11701-026-03816-5 (PMC13489991; doi:10.1007/s11701-026-03816-5)
Supplement: Supplementary file 1 [file 11701_2026_3816_MOESM1_ESM.docx]

| Time point  (month) | Group | Adjusted mean  (cm^3^) | Adjusted difference vs control  (IC 95%) | Holm-adjusted p-value |
| --- | --- | --- | --- | --- |
| THIGH - RIGHT | | | | |
| 1 | **Control** | 7463.851 | - | 0.958 |
| 1 | **Treatment** | 7606.529 | 142.678  [-256.1061 541.462] |  |
| 3 | **Control** | 7780.353 | - | 0.965 |
| 3 | **Treatment** | 7788.641 | 8.287637  [-363.2639 379.8392] |  |
| SHANK - RIGHT | | | | |
| 1 | **Control** | 3671.001 | - | 0.124 |
| 1 | **Treatment** | 4101.202 | 430.2012  [-21.9681 882.3706] |  |
| 3 | **Control** | 3809.066 | - | 0.526 |
| 3 | **Treatment** | 3921.023 | 111.9569  [-237.4231 461.337] |  |
| THIGH - LEFT | | | | |
| 1 | **Control** | 7369.789 | - | 0.376 |
| 1 | **Treatment** | 7083.14 | -286.649  [-716.0148 142.7169] |  |
| 3 | **Control** | 7670.69 | - | 0.740 |
| 3 | **Treatment** | 7602.173 | -68.51701  [-477.6218 340.5878] |  |
| SHANK - LEFT | | | | |
| 1 | **Control** | 3617.188 | - | 0.888 |
| 1 | **Treatment** | 3692.95 | 75.76181  [-120.6248 271.3204] |  |
| 3 | **Control** | 3776.707 | - | 0.818 |
| 3 | **Treatment** | 3803.012 | 26.30518  [-199.8977 252.508] |  |

**Supplementary Table 1.** Adjusted 1-3 months thigh and shank volume difference
