# Supplementary material for: Prevention of lymphedema after extended pelvic lymphadenectomy using diosmin, bromelain, melilotus, and hesperidin: results from a prospective randomized study (PRELYNE trial)
Source: J Robot Surg. 2026 Aug 19;20(1):856. doi: 10.1007/s11701-026-03816-5 (PMC13489991; doi:10.1007/s11701-026-03816-5)
Supplement: Supplementary file 2 [file 11701_2026_3816_MOESM2_ESM.docx]

|  | **Total N=98** | **Control group N=49** | **Treatment group N=49** | **p** |
| --- | --- | --- | --- | --- |
| **Thigh volume Δ% 3 months-1 month** |  |  |  |  |
| right | 5.57 (20.04) | 7.13 (25.35) | 3.86 (11.84) | 0.9 |
| left | 7.01 (28.38) | 4.54 (8.77) | 9.72 (40.07) | 0.64 |
| **Shank volume Δ% 3 months-1 month** |  |  |  |  |
| right | 5.27 (24.70) | 4.96 (14.55) | 5.62 (32.64) | 0.31 |
| left | 4.92 (13.44) | 4.19 (12.37) | 5.73 (14.64) | 0.66 |
| Mean (s.d.) |  |  |  |  |

**Supplementary Table 2.** Limbs 1-3 months Δ% variation.
